# Refining Unfavorable Vaginal Microbial Community in Infertile Women Subjected to Precision Probiotic Intervention: An Exploratory Single-Arm, Prospective, Open-Label, Interventional Study

**DOI:** 10.3390/microorganisms13030547

**Published:** 2025-02-28

**Authors:** Giovanna Cocomazzi, Viviana Contu, Silvia De Stefani, Lino Del Pup, Matteo Buccheri, Monica Antinori, Lodovico Parmegiani, Daniele De Ruvo, Francesco Marino, Edy Virgili, Christine Allen, Simone Palini, Walter Ciampaglia, Matteo Cerboneschi, Domenico Baldini, Giorgio Maria Baldini, Valerio Pazienza

**Affiliations:** 1 Division of Gastroenterology, Fondazione IRCCS-Casa Sollievo della Sofferenza, 71013 San Giovanni Rotondo, FG, Italy; g.cocomazzi@operapadrepio.it; 2 Integrative Medicine Unit, Humanitas Gradenigo, Corso Regina Margherita 8/10, 10153 Torino, TO, Italy; 3 Clinica Nuova Ricerca, Via Settembrini 17/h, 47923 Rimini, RN, Italy; silviadestefani@ymail.com; 4 Gynecological Endocrinology and Fertility, University Sanitary Agency Friuli Central (ASUFC), Via San Valentino 18, 33100 Udine, UD, Italy; info@delpupginecologia.it; 5 New Fertility Group, Via Portuense ROMA, 00149 Rome, RM, Italy; matteobuccheri@gmail.com; 6 Raprui Clinic, Via Timavo, 2, 00195 Roma, RM, Italy; monica.antinori@raprui.com; 7 Next Fertility GynePro, NextClinics International, Via T. Cremona 8, 40137 Bologna, BO, Italy; lodovico.parmegiani@gynepro.it (L.P.); walter.ciampaglia@gynepro.it (W.C.); 8 Gynaecology, Obstetrics and Reproductive Medicine Affidea Promea, Via Menabrea 14, 10126 Torino, TO, Italy; daniele.deruvo@gmail.com; 9 Association for Research on Integrative Oncological Therapies (ARTOI), Via Ludovico Micara 73, 00165 Roma, RM, Italy; dr.fmarino@gmail.com; 10 School of Biosciences and Veterinary Medicine, University of Camerino, 62032 Camerino, MC, Italy; info@edyvirgili.it; 11 Reproductive Laboratory Services, LLC, 3500 S. Dupont Highway, Dover, DE 19901, USA; ca@fertilityconsultancy.com; 12 Ospedale “Cervesi” di Cattolica-AUSL Romagna, Via Ludwig Van Beethoven, 1, 47841 Cattolica, RN, Italy; simonepalini@yahoo.it; 13 NEXT Genomics S.r.l, Via Madonna del Piano 6, 50019 Sesto Fiorentino, FI, Italy; matteo@personalnext.it; 14 IVF Center, Momò Fertilife, Via Cala dell’Arciprete, 76011 Bisceglie, BT, Italy; dbaldini@libero.it (D.B.); gbaldini97@gmail.com (G.M.B.)

**Keywords:** vaginal microbiome, probiotics, infertility

## Abstract

Background and aims: Vaginal microbiomes have been classified into five different general categories, termed Community State Type (CSTs), with CST-III and CST-IV often associated with vaginal dysbiosis which makes women more prone to recurrent infections and assisted reproductive technology (ART) failure. Since a healthy microbiome is one of the key steps for successful reproduction, we investigated the impact of modulating the vaginal microbiota through the oral administration of probiotic formula consisting of a consortium of vaginal-specific lactobacilli and prebiotics (Personal Flora 2^®^). Methods: We recruited 32 women who had previous failed IVF cycles and were scheduled to undergo ART. We examined the composition of the vaginal microbiota before and after oral probiotic supplementation using 16S ribosomal RNA (rRNA) sequencing technology. Results: Our data show a noticeable modulation of the vaginal microbiome upon probiotic supplementation. In particular, precision probiotic intervention lowers the species diversity, favoring the dominance of *Lactobacillus* (*p* = 0.015) and *Bifidobacterium* (*p* = 0.000) whilst decreasing the percentage of *Atopobium* (*p* = 0.003), *Gardnerella* (*p* = 0.022), and *Prevotella* (*p* = 0.000). Conclusions: Although CST-III and CST-IV are generally considered detrimental, gynecologists should not refrain from performing IVF in these women if they have been previously subjected to a consortium of precision probiotics treatments, as the administration of specific probiotics reduces the presence of pathogenic bacteria promoting the increase in lactobacilli associated with a healthy vaginal ecosystem, which could impact pregnancy success.

## 1. Introduction

Vaginal microbiota under balanced conditions display a lower bacterial diversity than the gut microbiome [1] because of lactobacilli’s predominance, which is crucial in preserving the equilibrium of this intricate environment. Therefore, the reproductive system’s equilibrium and well-being are significantly influenced by the vaginal microbiome. According to recent evidence, the vaginal microbiome’s composition affects a number of health-related factors, including fertility, infections and health. Every woman has a unique microbiota that might be altered depending on her lifestyle and hormonal fluctuations at different phases of life [2]. Depending on the type and quantity of lactobacilli or other species colonizing the female genital tract, vaginal microbiomes have been divided into five broad categories, called Community State Type [3]. CST-I is mainly dominated by *Lactobacillus crispatus*; while *Lactobacillus gasseri* dominates CST-II; *Lactobacillus iners* dominates CST-III; and a consortium of obligate and facultative anaerobe bacteria from the genera *Gardnerella*, *Atopobium*, *Mobiluncus* and *Prevotella* dominate CST-IV [3]. *Lactobacillus jensenii* dominates CST-V [3]. Women have varied bacterial communities, but within each of these categories, there is also various intersubject variability. Numerous studies have demonstrated that both aerobic and anaerobic microbes make up the vaginal microbiota of healthy women. In addition, the composition of the vaginal flora varies depending on the stages of female development [4]. In contrast, the microbial population is very stab+le during menopause; experiencing few changes in terms of balance between the various species. The vaginal microflora is subject to frequent variations even during the menstrual period; in fact, particular species may predominate over the others in rotation [5]. The primary species that predominate in women who are of childbearing age are *Lactobacillus gasseri*, *Lactobacillus crispatus*, *Lactobacillus jensenii* and *Lactobacillus iners* [6]. Lactobacillus species—which are invaluable allies in the defense of the female genital mucosa—predominate in the healthy vaginal microbiota through a variety of processes, including the generation of bacteriocines and the production of lactic acid, ensuing low pH and thus preventing the growth and adhesion of the harmful ones. Lactic acid is released when vaginal lactobacilli ferment glucose and maltose that are derived from the glycogen that is abundant in the vaginal epithelium [7]. By preventing the growth of certain harmful microorganisms such as *Candida albicans*, *Gardnerella vaginalis*, *Neisseria gonorrhoeae*, *Chlamydia trachomatis*, and herpes simplex virus type 2 (HSV-2), the synthesis of this compound contributes to maintaining an acidic pH within the environment [8]. According to recent research, women with bacterial vaginosis are more susceptible to contracting HIV infection compared to women with a healthy microbiota [9]. It has been observed that women with a lactobacillus-dominated microbiota are able to inactivate HIV due to the anti-inflammatory action of lactic acid, and/or may be less likely to transmit the virus to their unborn child after childbirth [9,10]. Additionally, through the antagonistic action of lactic acid, lactobacilli reduce the HSV’s ability to adhere to the host cell membrane, preventing both entry and viral reproduction [11]. Bacterial vaginosis develops when lactobacilli are significantly reduced due to vaginal dysbiosis [3]. The loss of lactobacilli results in a shift from a healthy flora state to bacterial vaginosis, which is linked with negative reproductive outcomes, such as implantation failure, preterm birth and miscarriage [12]. The bacteria most commonly related with preterm birth are *Ureaplasma*, *Mycoplasma*, *Atopobium*, *Prevotella*, *Gardnerella vaginalis* and *Fusobacterium* [13]. Their presence often does not cause clinical symptoms, but it can cause a chronic infection resulting in the release of pro-inflammatory cytokines, triggering preterm rupture of membranous and preterm birth. Currently, infertility work-up and treatment do not include screening and treatment to restore the vaginal microbiota. Recent studies highlight the link between vaginal microbiota composition and pregnancy outcomes in patients undergoing ART. Numerous factors, such as maternal age, follicle-stimulating hormone (FSH) and anti-mullerian hormone (AMH) levels, inadequate ovarian reserve, the quality of both male and female gametes, psychological factors, high BMI and genetic factors can affect the result of assisted reproduction treatments, according to data from the literature [14,15,16]. About one-third of patients undergoing IVF become pregnant, while more than 60% of cases end in failure [17]. Current research looks at the potential presence of additional variables that could have a negative impact on ART results, including the vaginal microbiome. It has been noted that the pregnancy rates of women receiving ART have been adversely affected by microorganisms linked to vaginal dysbiosis, including *Gardnerella* and *Atopobium* [18]. About 50% of women who have undergone ART with low abundance of endometrial lactobacillus experienced poor implantation rates and poor reproductive outcomes, according to Moreno et al. [19]. Probiotic therapy has been considered as one of the novel approaches used to treat and prevent vaginal infections. The effectiveness of probiotics alone or in combination for the treatment of BV has been the subject of numerous investigations. Several studies have demonstrated that orally administered lactobacilli are able to migrate from the intestine to the vagina, decreasing vaginal colonization by urogenital pathogens [20,21,22,23] improving systemic immune responses through local immunomodulation [24]. Precision probiotics are more specifically formulated based on analyzing the vaginal microbiota through advanced techniques such as DNA sequencing, and it is possible to determine whether beneficial bacterial strains such as lactobacilli are missing and whether pathogenic species are present. In this way, precision probiotics can be chosen according to the patient’s individual needs. In addition, precision probiotics are designed to be tailored to their needs; taking into account health conditions, medical history and environmental factors affecting the vaginal microbiota. These approaches are still in the research and development phase, providing a much more targeted and personalized solution than traditional generic probiotic treatments. Based on these concepts, the main aim of this study was to assess the ability of specific probiotic formulation derived from a previous study where we detected a cluster of bacteria which was predictive of favorable pregnancy outcomes on shifting the vaginal microbiota profile in women undergoing ART.

Here, we provide the findings of the effects of precision probiotic formula treatment on the vaginal microbiota profile of infertile women undergoing ART.

## 2. Materials and Methods

### 2.1. Study Design and Probiotic Intervention

**Figure 1:**
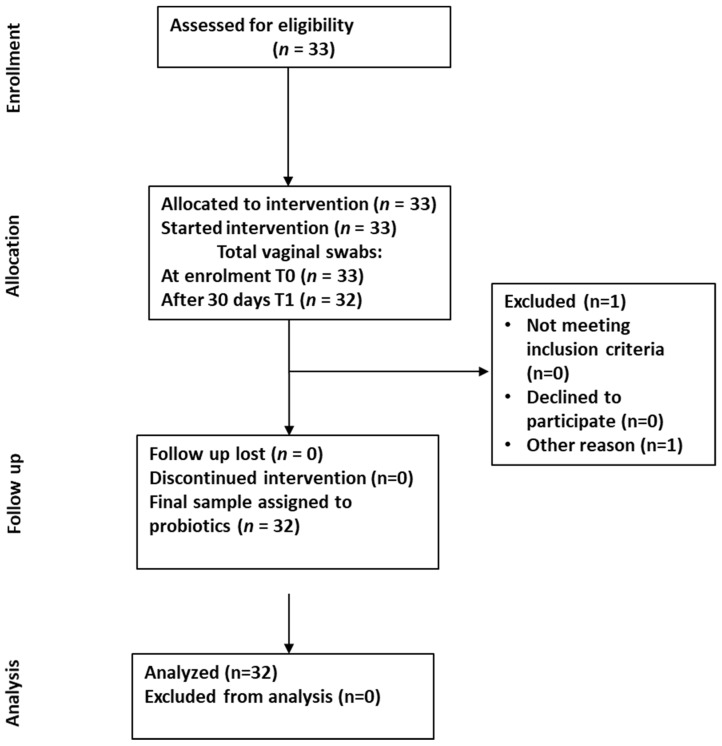
Flow diagram for a single-arm study of a probiotics treatment.

The study cohort comprised 32 Caucasian patients aged from 29 to 49 years who had infertility issues with a history of recurrent miscarriage and/or implantation failure. They received a specifically formulated probiotic mixture (Personal Flora 2^®^) for 30 days, prior to undergoing the IVF procedure. Figure 1 shows the flow diagram of the single-arm study design. Participants consumed probiotic sachets orally at least 20 min before breakfast at the dose of 1 sachet for 30 consecutive days. Each sachet contained not less than 10 billion colony forming units (CFU) of *L. gasseri* (SGL09), *L. acidophilus* (SGL11), *L. casei* (SGL15), *B. Breve* (SGB01), *L. crispatus* (6272) with FOS (Fructooligosaccharide, 1 g) and acacia fiber (1 g). The dose of each probiotic strain had 2 × 10^9^ CFU, and the total weight was 3 g. Vaginal swabs were collected at the baseline (T0 = Day 0) and after probiotics administration (T1 = Day 30). All participants filled out a questionnaire with their information such as body mass index (BMI), alcohol use, smoke, infection, dietary habits and medical histories.

### 2.2. Ethics Statement

All patients signed and provided an informed consent related to the present study under the approval of the Human Ethics Committee of Nuova Ricerca Hospital (approval number C.E. 002\2023) and from IRCCS “Casa Sollievo della Sofferenza Hospital” (Prot.n.125/CE).

### 2.3. Sample Collection and DNA Extraction

Vaginal swabs were stored for each study participant in a sterile tube with a DNA stabilization buffer (Copan, Brescia, Italy n. cat. 608C), and the tubes were centrifuged for 10 min at 7500 rpm. Following the manufacturer’s instructions, total genomic DNA was extracted using the QIAamp DNA blood and tissue kit (Qiagen, Milan, Italy, Cat. No. 69504). After the isolation process was completed, the concentration and purity of the DNA were examined, and it was frozen at −80 °C for storage.

### 2.4. Sequencing of Bacterial 16S rRNA Gene

The V3–V4 region was amplified, starting from 5 ng of each DNA using KAPA HiFi HotStart ReadyMix (Roche Diagnostics, Milan, Italy, Cat N° 07958935001) as previously described [25] with the following primers with Illumina adapters: 5′-TCGTCGGCAGCGTCAGATGTGTATAAGAGACAGCCTACGGGNGGCWGCAG; and the reverse primer, 5′-GTCTCGTGGGCTCGGAGATGTGTATAAGAGACAGGACTACHVGGGTATCTAATCC [26]. Library preparation was based on equimolar concentrations for 2 × 300 paired-end sequencing using the MiSeq Reagent Kit v3 (600 cycle) (Illumina, Milan, Italy, Cat N° MS-102-3003), and samples were barcoded using the Nextera XT Index Kit (Illumina, Milan, Italy, Cat N° FC-131-1002). DNA samples were quantified using a Qubit 3.0 Fluorometer (Invitrogen, Carlsbad, CA, USA). The taxonomic analysis of FASTq files generated by the MiSeq instrument was conducted using the 16S Metagenomics GAIA v.2.0 tool. FASTq files with the code E-MTAB-14653 were uploaded to ArrayExpress. Quality control (i.e., trimming, clipping, and adapter removal) was based on FastQC and BBDuk and mapped with BWA-MEM against a custom database (based on NCBI) in order to ascertain the taxonomic profile of each sample and analyze the microbiota profile before and after probiotic treatment for 30 days.

### 2.5. Statistical Analysis

The mean ± standard deviation (SD), median and interquartile range (i.e., first–third quartiles), and observed frequencies (and percentages) for continuous and categorical variables, respectively, were used to report the patients’ demographic and clinical features. The DESeq2 analysis was used to determine the composition and abundance of the vaginal microbiota (i.e., mean relative abundance %) at the phylum, family, genus, and species levels between patients at baseline and following probiotic therapy. Significant results were defined as *p* < 0.05 and FDR < 0.05. The associations between vaginal microbiota and age, height, weight, and BMI were examined using Spearman’s correlation. The software calculated the relative abundance of bacterial species for each sample and also gave the Chao1 richness estimation and the Shannon alpha diversity index. To compare ranks, the Mann–Whitney test was performed. A *p*-value of less than 0.05 was deemed statistically significant.

## 3. Results

### 3.1. Patients’ Demographic, Anthropometric and Clinical Characteristics

In our study, a total of 33 patients were enrolled. Of these, 32 completed oral intake of probiotics for 30 days. One patient was excluded due to the lack of vaginal samples at the specific time points (T1). In Table 1 the characteristics of the patients are summarized, including: body mass index (BMI), alcohol use, smoke, dietary habits, physical activity, medical histories (previous pregnancy, comorbidities, medical drugs consumption).

### 3.2. Vaginal Microbial Richness and Diversity upon Precision Probiotic Treatment

**Figure 2:**
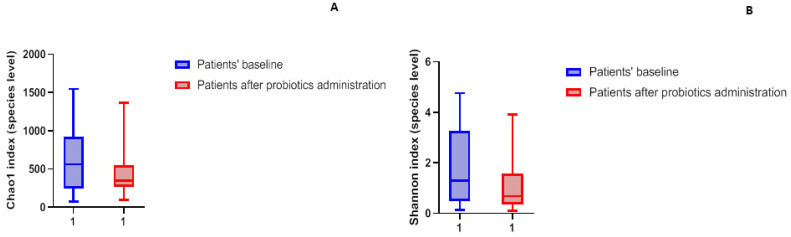
Box-plots of Chao1 index of species richness (**A**) in patient’s baseline and after administration of probiotics; box-plot of Shannon index (**B**).

No statistically significant difference was found for Chao1 (species richness) index (Figure 2A) between samples at baseline and samples after oral administration of probiotics. In contrast, the Shannon index, which accounts for the number and relative abundance of species within each sample, differed significantly (*p* ≤ 0.05) (Figure 2B) after administration of probiotics. It is important to note that the intervention of probiotics lowers the microbial diversity, consistent with the concept that healthy vaginal microbiota, unlike intestinal microbiota, is characterized by low microbial diversity. In contrast, an unhealthy microbiota is characterized by an increased diversity due to the presence of pathogenic species causing a reduction in lactobacilli.

### 3.3. Dynamic Changes in the Composition of Vaginal Microbiota in Women upon Precision Probiotic Intervention

**Figure 3:**
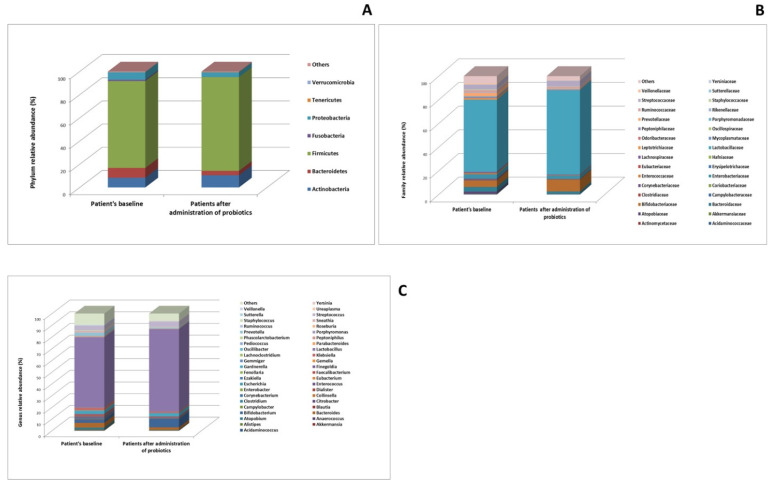
The vaginal microbiota composition pre-intervention (baseline) and post-intervention (after administration of probiotics). (**A**) abundance at the phylum level; (**B**) abundance at the family-level; (**C**) genus-level abundance.

Vaginal microbial abundance levels at baseline and following probiotic delivery reported by phylum, family and genus is reported in Figure 3. At phylum level (Figure 3A), following oral probiotic intervention, there was a significant increase in the abundance of Actinobacteria (8.35% vs. 10.48%) and Tenericutes, (0.04% vs. 0.25%). At the family level (Figure 3B), we found that the abundances of *Bifidobacteriaceae* (5.43% vs. 9.97%) and *Mycoplasmataceae* (0.018% vs. 0.24%), significantly increased after the intervention, while the abundances of *Atopobiaceae* (1.65% vs. 0.022%), *Enterobacteriaceae* (3.75% vs. 2.80%) and *Prevotellaceae* (2.73% vs. 0.66%) was significantly decreased. At the genus level (Figure 3C), it was discovered that probiotic-treated individuals had significantly higher levels of *Bifidobacterium* (2.49% vs. 7.09%) as compared to the baseline.

**Figure 4:**
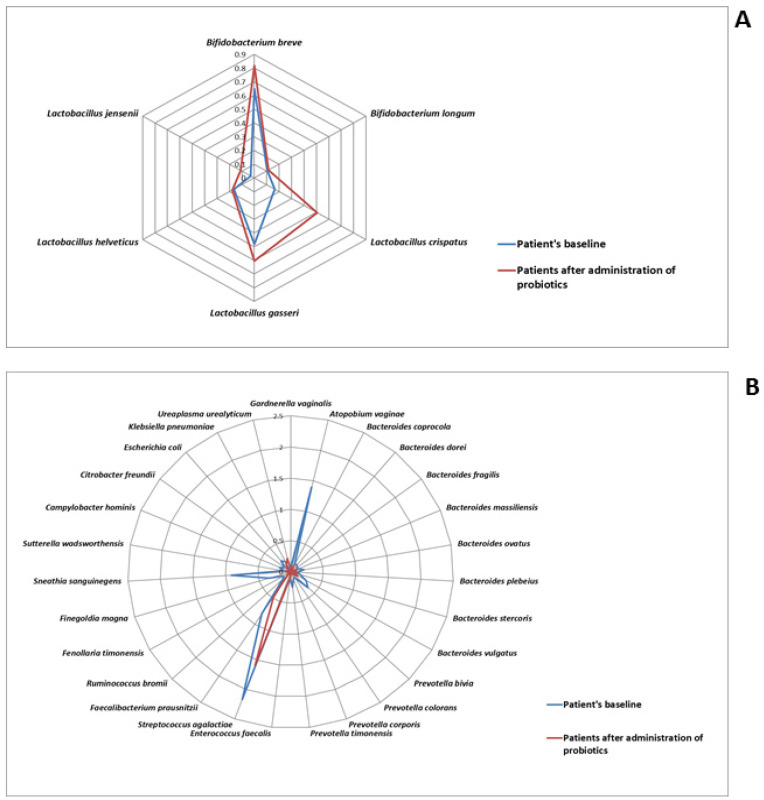
Radar chart of the species-level in patients’ baseline and patients after administration of probiotics. (**A**) Representation of the bacteria associated with healthy vaginal ecosystem; (**B**) representation of the species associated with infertility.

Additionally, *Atopobium* (1.61% vs. 0.01%) and *Prevotella* (2.56% vs. 0.62%) were significantly under-represented. *Atopobium vaginae* (1.40% vs. 4.19 × 10^−5^%), *Prevotella bivia* (0.36% vs. 0.07%) and *Citrobacter freundii* (0.12% vs. 0.03%) are among the pathogenic species linked to idiopathic infertility that significantly decreased in all patients following probiotic intervention, as shown graphically in Figure 4. The percentage of lactobacillus and *Bifidobacterium* species rises following treatment. Specifically, the prevalence of *Bifidobacterium breve* (0.65% vs. 0.81%) significantly increases. *Lactobacillus crispatus* (0.16% vs. 0.50%) increases from the baseline; the result was statistically significant based on the *p*-value threshold; however, it did not remain significant after correction for multiple comparisons using the false discovery rate (FDR).

**Figure 5:**
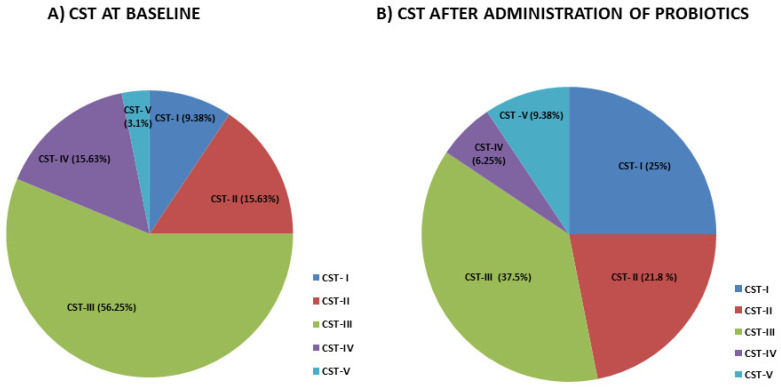
Characteristics of the types of community structures of the vaginal microbiome (CST). The proportion of the Community State Types (CSTs) in patients’ baseline (**A**) and after administration of probiotics (**B**).

As for the CSTs classification, the vaginal microbiota of the 32 patients were identified as follows (Figure 5): CST-I, 9.38% (3/32), CST-II, 15.63% (5/32), CST-III, 56.25% (18/32), CST-IV, 15.63% (5/32) and CST-V 3.1% (1/32). All were present in patient’s baseline. Comparing the CST in patients after administration of probiotics, we observed an increase of CST-I, 25% (8/32), CST-II, 21.8% (7/32) and CST-V, 9.38% 3/32; while there was a decreased number of patients having CST-III, 37.5% (12/32) and CST-IV, 6.25% (2/32); confirming a shift through a healthier vaginal microbiota profile upon treatment.

### 3.4. Correlation Analysis Between Vaginal Microbiota Profiles and Clinical Parameters

**Figure 6:**
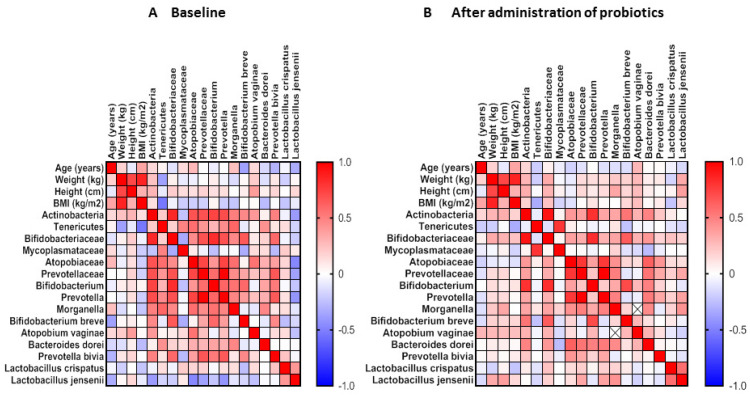
Relationships between age, height, weight and BMI metrics, and vaginal microbiota composition (phylum, family, genus and species) in infertile patients receiving ART at baseline and following oral probiotic treatment.

Following the identification at each taxonomic level, the bacteria were compared to the patients’ BMI, weight, height, and age at baseline and following probiotic therapy. Spearman’s correlation analyses reveal some associations before and after the oral probiotics intervention (Figure 6). In the patients’ baseline at the phylum level, Tenericutes were found to negatively correlate with weight and BMI. At species level, *Bifidobacterium breve* showed an inverse correlation with age, while *Atopobium vaginae* were positively associated with height; after treatment, height was positively correlated with *Lactobacillus jensenii*.

## 4. Discussion

Infertility is a medical issue affecting millions of people worldwide. WHO estimates that one in six people suffers from infertility, which affects roughly 17.5% of the world’s population. Male and female infertility can be caused by a number of factors but attempts to link vaginal microbiota dysbiosis to female infertility are still in their infancy. Reproductive success is known to be correlated with a healthy vaginal microbiota, which is dominated by lactobacilli. On the other hand, bacterial vaginosis and the disequilibrium of the vaginal microbiota are associated with the presence of several pathogenic species, including *Gardnerella*, *Atopobium*, *Prevotella*, *Bacteroides*, *Peptostreptococcus*, *Mobilincus*, *Sneathia*, *Leptotrichia*, *Mycoplasma* and *Clostridiales* [27]. Precision probiotics help women with infections or undergoing assisted reproductive techniques (ART) by generating a favorable environment during the conception stage, which is crucial for the formation of healthy pregnancies. Probiotics can enhance the vaginal microbiota’s health through a number of ways, including bacterial competition and vaginal barrier impact. In particular, lactobacilli can create colonies that stick to the vaginal epithelium’s cells using glycoproteins, creating a physical barrier that stops pathogens from adhering [28]. They can do this by generating substances that inhibit adhesion, such as lactic acid, organic acids, and bacteriocins. By stimulating the production of immunoglobulin A, probiotic lactobacilli also have the ability to counteract infections through immunomodulatory action. In particular, the presence of *Lactobacillus jensenii* and *Lactobacillus crispatus* reduces the quantity of proinflammatory cytokines released by pathogenic microorganisms such as *Gardnerella vaginalis* [29] including IL-1α and IL-8 [30]. Our results shown that precision probiotics intervention decreases the abundance of pathogenic species. Specifically, we observed that *Atopobium vaginae*, *Prevotella bivia* significantly decreased while species correlated with healthy vaginal microbiota such as *Lactobacillus crispatus* and *Bifidobacterium* increased. Remarkably, the percentage of patients with Community State Types changes upon probiotics intervention. Specifically, the percentage of patients with CST III and CST IV decreased. Conversely, following probiotic therapy, the percentage of patients with CST-I (9.38 vs. 25%), CST-II (15.63% vs. 21.8%) and CST-V (15.63% vs. 21.8%) increased. Several taxa displayed significant positive or negative correlations with the patients’ age, height, weight, and BMI when the vaginal microbiota profile was compared to these variables. At the baseline, *Bifidobacterium breve* showed an inverse correlation with age. According to previous studies, there is a decline in the gut microbiota of *Bifidobacterium* species in adulthood, compared to younger aged. This decline could be due to a physiological reduction or lifestyle changes, such as the use of antibiotics or drugs which are consumed in increasing quantities as an adult [31]. Even though the vagina contains few *Bifidobacterium*, these bacteria are essential as it was demonstrated to reduce inflammation, and increase immunity and the process of folliculogenesis, and to regulate the levels of the sexual hormones FSH and LH in women with polycystic ovary syndrome (PCOS) [32,33,34], indicating a potential positive impact on oocyte maturation and quality [35]. Furthermore, it has been observed that the success rate of couples undergoing ART is increased by approximately two-fold when *Lactobacillus crispatus* is abundant [36]. Pathogenic bacteria such *Atopobium* and *Prevotella*, which are known to cause vaginal infections, generate pro-inflammatory cytokines, and hence decrease fertility, were significantly reduced in our study following probiotic therapy. Although we are aware that single-arm trials have limitations since they do not adhere to the principles of randomization and blinding in terms of scientific rigor, they still incorporate principles of control, balance and replication, making the design scientifically reasonable. Compared to randomized controlled trials, single-arm trials require fewer sample sizes and have shorter trial durations, which can help save costs. As a result, single-arm trial designs have emerged as one of the methods to address these issues. Single-arm trials are commonly applied to study advanced-stage cancer, rare diseases, emerging infectious diseases, new treatment methods and medical devices.

Our results indicate a reduction in microbial diversity in the vaginal microbiota upon probiotic administration and a rise in lactobacilli and *Bifidobacterium*. Although higher microbial diversity is generally associated with greater microbial health in many contexts, for the vaginal microbiota, lower diversity is considered positive; specifically, when it reflects a predominance of beneficial species, such as *Lactobacillus* which are essential for maintaining a healthy vaginal environment conducive to fertility. According to a study by Baud et al. [37], it was observed that a vaginal microbiota dominated by *Lactobacillus*, and thus characterized by low diversity, is associated with positive pregnancy outcomes; whereas higher diversity is associated with the risk of preterm delivery. The lower diversity following probiotic supplementation could indicate that probiotics promoted a more homogeneous microbial community focused on species that promote vaginal health, creating a more favorable environment for conception and reducing the likelihood of infections that could compromise ART treatments. Probiotics taken orally have the ability to colonize the vagina through specific processes. Direct colonization using the same colonization mechanism as uropathogenic bacteria is one of the proposed processes [38]. Because the gastrointestinal and vaginal tracts are physiologically connected, some strains can pass via the intestine, enter the rectum, and then enter the vagina [38]. Once lactobacilli enter the vagina, the availability of glycogen—in which the vaginal epithelium is especially rich—provides an environment that is conducive to colonization. They adhere to the vaginal epithelium with ease. Adhesion can also be enhanced by adhesins, which are glycoproteins and carbohydrates that bind to glycolipid receptors [28]. Therefore, probiotic strains may regulate the vaginal environment inside the vaginal district by producing antimicrobials, coaggregating, and engaging in adhesive competition. Another theory is that commensal bacteria cause B cells in the gut to produce IgA, which is coated by the bacteria and promotes the creation of biofilm, which travels through the bloodstream to the vagina and permits colonization [39]. Indirect translocation, or the bacterial strains’ synthesis of metabolites such as SCFA that operate in other areas such as the vaginal immune system, is another theory that has been put forth [39]. The final mechanism involves the action of the estrobolome; a complex of gut microorganisms that includes lactobacilli [39]. These microorganisms are able to metabolize estrogen, which enters the vaginal epithelial cells through the bloodstream and produces glycogen which the lactobacilli use to produce lactic acid; this is crucial for preserving vaginal health [39].

By moving to a favorable CST dominated by lactobacilli, women who also had a CST-IV that was deemed unfavorable because of the presence of anaerobic bacteria saw a noticeable improvement. Nevertheless, additional research is required to determine whether probiotic use significantly affects success rates. In order to determine whether probiotic use improved steroidogenesis and ovarian reserve quality, we will need to expand the number of participants, extend probiotic intake to observe the long-term effect and integrate additional data such as hormone dosage of FSH, LH and AMH to obtain data on pregnancy rates and determine whether probiotic use improved ovarian reserve quality and steroidogenesis.

## 5. Conclusions

An increasing number of couples around the world struggle with infertility, leading to a devastating impact on psychological and physical well-being. While some of the causes of infertility are known, many are still unknown. One of the reasons for infertility issues could be vaginal microbiota dysbiosis, particularly when certain harmful species numerically displace other healthy species. Our data show that by integrating bacteria associated to favorable pregnancy outcomes, it is possible to improve the vaginal microbiota profile in women undergoing ART, as demonstrated by the reduction of pathogenic species and an increase of percentage of patients displaying a shift through a healthier CST. Although single-arm trials have limitations, they still incorporate principles of control, balance and replication, making them an efficient method to study new treatment methods and medical devices. Precision probiotics use may present a feasible and promising approach to safely and non-invasively improving fertility outcomes.

## Figures and Tables

**Table 1:** Baseline characteristics of enrolled infertile patients.

Variable	Category	All Patients (N = 32)
*Anthropometric characteristics*	**---**	**---**
Age at enrollment (years)	Mean ± SD	38.3 ± 5.1
BMI at enrollment (kg/m^2^)	Median [IQR]	22.3 [20.2–24.5]
Height (cm)	Median [IQR]	166.0 [160.0–170.0]
Weight (kg)	Median [IQR]	60.0 [51.0–68.0]
*Medical history*	**---**	**---**
Previous pregnancies—N (%)	Yes	4 (12.5)
	No	14 (43.8)
	Miscarriage	4 (12.5)
	Not reported	10 (31.3)
Comorbidities—N (%)	Thyroid function disorders	6 (18.8)
	Others	7 (21.9)
	No	7 (21.9)
	Not reported	14 (43.8)
Medical drugs consumption—N (%)	Yes	15 (46.9)
	No	5 (15.6)
	Not reported	12 (37.5)
*Lifestyle info*	**---**	**---**
Physical activity—N (%)	Yes	8 (25.0)
	No	8 (25.0)
	Not reported	16 (50)
Smoking status—N (%)	Yes	3 (9.4)
	No	28 (87.5)
	Not reported	1 (3.1)
Alcohol consumption—N (%)	Habitually	1 (3.1)
	Occasionally	15 (46.9)
	No	15 (46.9)
	Not reported	1 (3.1)
Diet—N (%)	Mediterranean	7 (21.9)
	Hypoglucidic	3 (9.4)
	Free/omnivorous	17 (53.1)
	Vegetarian	2 (6.3)
	Not reported	3 (9.4)

## Data Availability

The data presented in this study are openly available in ArrayExpress under the code E-MTAB-14653. [ArrayExpress] [https://www.ebi.ac.uk/biostudies/arrayexpress] [E-MTAB-14653].
